# Museum material reveals a frog parasite emergence after the invasion of the cane toad in Australia

**DOI:** 10.1186/1756-3305-3-50

**Published:** 2010-06-10

**Authors:** Ashlie Hartigan, David N Phalen, Jan Šlapeta

**Affiliations:** 1 Faculty of Veterinary Science, University of Sydney, New South Wales 2006, Australia

## Abstract

**Background:**

A parasite morphologically indistinguishable from *Myxidium immersum *(Myxozoa: Myxosporea) found in gallbladders of the invasive cane toad (*Bufo marinus*) was identified in Australian frogs. Because no written record exists for such a parasite in Australian endemic frogs in 19^th ^and early 20^th ^century, it was assumed that the cane toad introduced this parasite. While we cannot go back in time ourselves, we investigated whether material at the museum of natural history could be used to retrieve parasites, and whether they were infected at the time of their collection (specifically prior to and after the cane toad translocation to Australia in 1935).

**Results:**

Using the herpetological collection at the Australian Museum we showed that no myxospores were found in any animals (*n *= 115) prior to the cane toad invasion (1879-1935). The green and golden bell frog (*Litoria aurea*), the Peron's tree frog (*Litoria peronii*), the green tree frog (*Litoria caerulea*) and the striped marsh frog (*Limnodynastes peronii*) were all negative for the presence of the parasite using microscopy of the gallbladder content and its histology. These results were sufficient to conclude that the population was free from this disease (at the expected minimum prevalence of 5%) at 99.7% confidence level using the 115 voucher specimens in the Australian Museum. Similarly, museum specimens (*n *= 29) of the green and golden bell frog from New Caledonia, where it was introduced in 19^th ^century, did not show the presence of myxospores. The earliest specimen positive for myxospores in a gallbladder was a green tree frog from 1966. Myxospores were found in eight (7.1%, *n *= 112) frogs in the post cane toad introduction period.

**Conclusion:**

Australian wildlife is increasingly under threat, and amphibian decline is one of the most dramatic examples. The museum material proved essential to directly support the evidence of parasite emergence in Australian native frogs. This parasite can be considered one of the luckiest parasites, because it has found an empty niche in Australia. It now flourishes in > 20 endemic and exotic frog species, but its consequences are yet to be fully understood.

## Background

Museum material is important in comparisons between historical and contemporary animal ranges in relation to environmental modifications [1]. Investigation of pathogens in museum material has been scarce, despite their offering a unique insight into parasite emergence throughout the museum's sampling period, often spanning several centuries [2].

Museum collections of amphibians have been used effectively in the global investigation into amphibian decline and historical emergence of external frog malformations [1,3]. Examination of museum material supported the 'out of Africa' origin of the amphibian chytrid (*Batrachochytrium dendrobatidis*) [4]. International trade of amphibians, that began in the mid-1930's, spread the chytrid which decimated amphibian populations worldwide [4]. Exotic introductions of myxosporeans are well known to the aquaculture industry. The myxosporean, *Myxobolus cerebralis *(Plehn 1905), which causes whirling disease in salmonid fish was introduced from Europe to 26 other countries [5] and has significantly impacted wild and farmed fish populations in North America significantly [6].

A myxosporean parasite *Myxidium immersum *(Lutz 1889) (Myxozoa; Myxosporea) was suggested to have been introduced to a wide spectrum of Australian native frogs during the 20^th ^century with the exotic cane toad (*Bufo marinus*) [7,8]. This assumption is based on the absence of any written record for myxosporean parasite in Australian endemic frogs in 19^th ^and early 20^th ^century [7]. *Myxidium immersum *was found in gallbladders of at least 12 species of *Litoria*, 4 species of *Limnodynastes*, and one each of *Mixophyes*, *Ranidella*, and *Uperoleia *in eastern Australia (Queensland, New South Wales) [7]. Moreover, a *Myxidium *sp. (myxospores closely resembling *Myxidium immersum*) infection was found in a green tree frog (*Litoria caerulea*) with hepatitis [9], and circumstantial evidence suggests that myxosporeans are important pathogens of frogs and despite being recognised as potential disease agents there has been little investigation into their threat status [10,11]. Currently, no life cycle for any frog myxosporean parasite has been elucidated [12]. It is speculated, that myxosporean parasites in frogs will require an invertebrate host, because the majority of myxosporean parasites in fish alternate between vertebrate and invertebrate hosts [13].

The historical emergence of *Myxidium immersum *in Australian amphibians can be effectively investigated by directly showing its absence in frogs prior to the well documented cane toad introduction into Queensland, Australia in 1935. To test the hypothesis that the emergence of myxosporeans in Australian frogs occurred after the introduction of the cane toad, we dissected frog voucher specimens from the Australian Museum to microscopically examine the presence of myxosporean stages before and after the cane toad translocation.

## Results

### No gallbladder myxospores found in frogs prior to 1935

**Figure 1:**
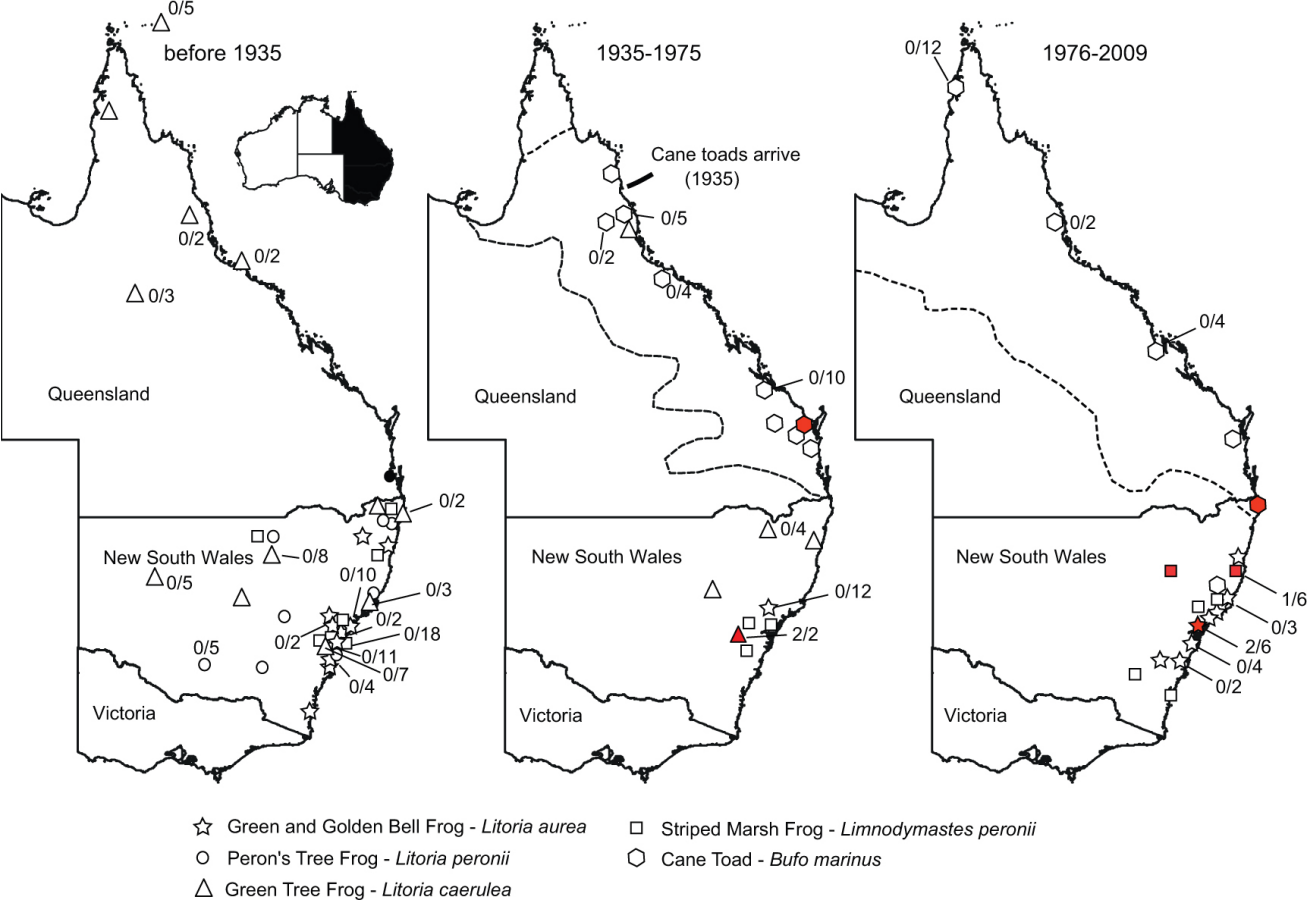
**Frog species collection sites in eastern Australia**. Maps of collection sites in eastern Australia with the distribution of frogs collected and catalogued at the Australian Museum were grouped according to their collection date. Locations of individuals with the presence of myxospores in gall bladders are indicated in red. Each host species is depicted with a different icon. When more than one specimen from a single location was examined, the positive/negative values are shown. The western border of the cane toad distribution is delineated by a broken line.

A catalogue of the Australian Museum, Sydney was queried for five frog species; the green and golden bell frog (*Litoria aurea*), the green tree frog (*Litoria caerulea*), the Peron's tree frog (*Litoria peronii*), the striped marsh frog (*Limnodynastes peronii*), and the introduced cane toad (*Bufo marinus*, syn. *Rhinella marina*). All specimens prior to 1935, for the green and golden bell frog (*n *= 27), the green tree frog (*n *= 48), the Peron's tree frog (*n *= 16) and the striped marsh frog (*n *= 24) representing Australian endemic species were retrieved (Additional file 1). No myxospores were found in the total of 115 voucher specimens collected before 1935. The museum specimens were from eastern Australia, mostly from New South Wales (Figure 1).

Assuming the test sensitivity and specificity to be perfect and the expected minimum prevalence to be 5%, the probability of observing zero positives in a sample of 115 frogs from before 1935 is 0.003. These results are sufficient to conclude that the population was free from this disease (at the expected minimum prevalence of 5%) at 99.7% confidence level. Assuming only 90% test sensitivity we have exceeded the 66 animals as the minimum number (p = 0.05) to be taken from a population with a negative result to consider the population as free of the disease (expecting minimum prevalence of 5%). Using 115 animals and test sensitivity of 90%, it is adequate to conclude, that the population is free from disease at the 99.5% confidence level.

No myxospores were found in a selection of museum specimens of the green and golden bell frog from New Caledonia (*n *= 29). The green and golden bell frog was introduced to New Caledonia in 19^th ^century [14].

### The earliest specimen positive for gallbladder myxospores is from 1966

**Figure 2:**
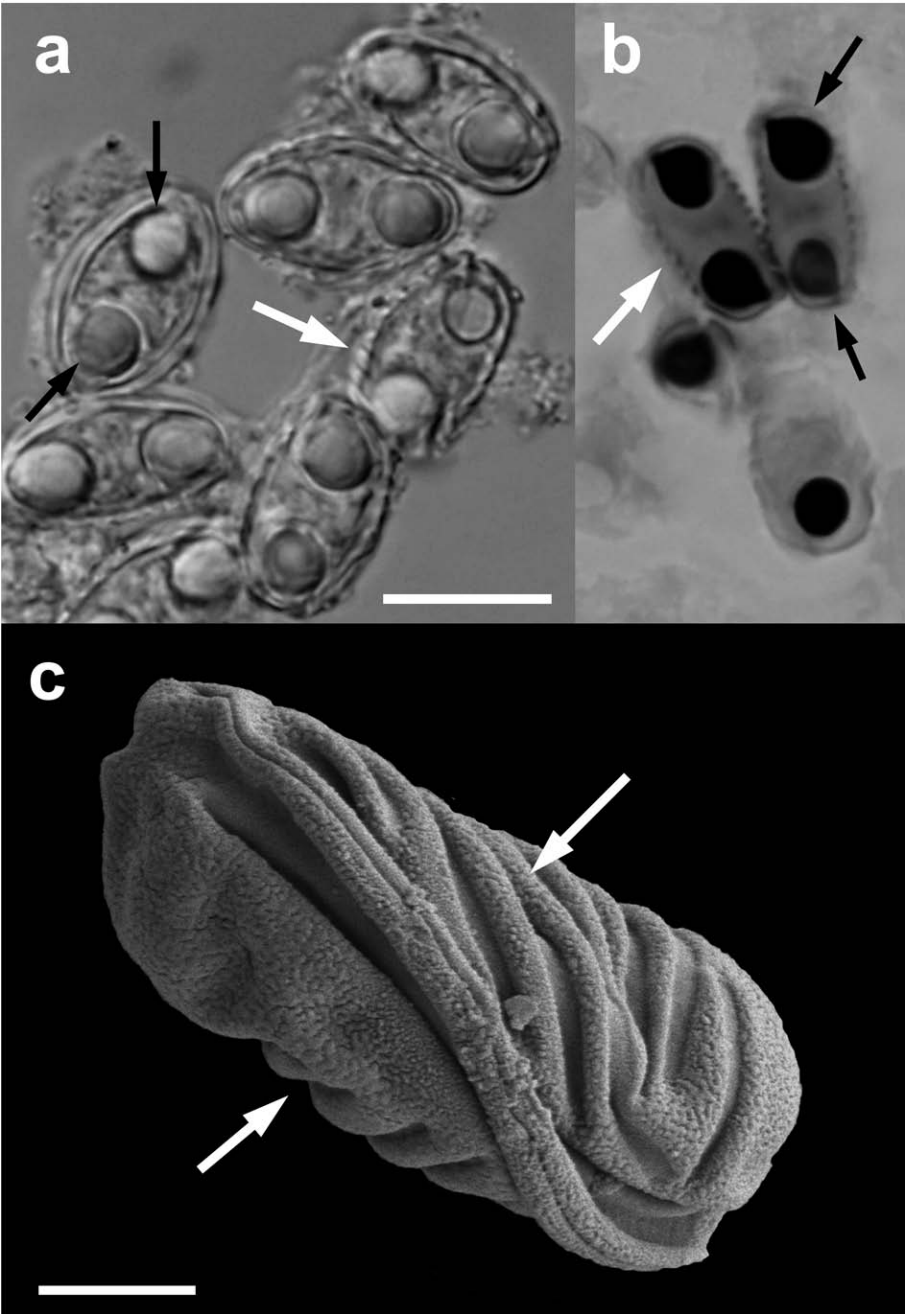
***Myxidium *cf. *immersum *spores recovered from the Australian Museum**. Light microscopy (A), histological section through the gallbladder stained with Giemsa (B) and scanning electron microscopy on black background. White arrows show the surface ridges and black arrows show two polar capsules per myxospore. Scale bar 10 μm (A, B), 2.5 μm (C); AM#158500 (A, C; *Bufo marinus*) and AM#162439 (B; *Limnodynastes peronii*).

In total, 112 voucher specimens and their catalogue records were recovered for frogs after 1935 (Figure 1, Additional file 1); the green and golden bell frog (*n *= 34), the green tree frog (*n *= 12), the striped marsh frog (*n *= 18) - representing Australian endemic species, and the introduced cane toad (*n *= 48). The earliest specimen positive for myxospores spores was from 1966 (green tree frog, New South Wales, AM#99574). Myxospores spores were found in eight (7.1%, 8/112) Australian frog vouchers in the post cane toad introduction period (Figure 1, 2; Additional file 1). The myxospore positive native frogs, the green and golden bell frog (AM#148744, AM#153967), the green tree frog (AM#99754, AM#99575) and the striped marsh frog (AM#158418, AM#162439), were from New South Wales. Two cane toads from New South Wales and Queensland were positive for myxospores (AM#158500, AM#59922), the earliest is from 1967.

Ultrastructurally, myxospores resembled *Myxidium *spores for which the number of ridges on the surface of *Myxidium *spores is a species diagnostic character. All examined myxospores from museum vouchers had 5-9 ridges on the myxospore surface (Additional file 1). Myxospore morphology was consistent under light microscopy (Figure 2A), histology (Figure 2B) and scanning electron microscopy (Figure 2C). The myxospores from individual host species were not identical in size, likely due to the effect of the ethanol or formalin fixation for an unknown period of time prior to being transferred into 70% ethanol at the Australian Museum. Nevertheless, the number of spore ridges, direction of the suture line and spore shape are compatible with *Myxidium immersum *[7,15]. This species was originally described from the cane toad specimens in Brazil, South America, and later it was re-evaluated and redescribed by Kudo and Sprague (1940) [15], who have provided the following measurements ranges for myxospores found in toad gallbladders: length and width, 11.8-14.2 × 7.5-10.0 μm, polar capsule length and width 3.5-4.5 × 3.3-4.2 μm, and recorded 7-9 transverse striations. Myxospores found in Australian frogs including the cane toad by Delvinquier (1986) [7] had the following characteristics: length and width 12.3-13.3 × 7.3-7.8 μm and 5-10 transverse striations (polar capsule measurements were not reported).

## Discussion

All vertebrates harbour a large diversity of parasites with often intricate life histories. For parasites whose life cycle comprises more than one specific host, the survival of such a parasite is dependent on sympatry and interaction with its host [16]. Removing a host from its natural environment where it has coevolved with its surroundings over a long historical trajectory may have major deleterious consequences for its parasites. However, parasites that do not need an additional host and can be sustained easily in the environment will be more likely to travel with their hosts especially if successively translocated multiple times across a great geographical distance, as experienced in the case of the cane toad. The cane toad was introduced around the world from its native range of Central and South America through the Caribbean into Queensland, Australia in 1935 [8]. The 101 translocated cane toads were bred and their progeny released as a biological control agent for a sugar cane industry suffering from beetle damage [8]. A single host lungworm parasite with a direct life-cycle (*Rhabdias pseudosphaerocephala*) is endemic to the cane toad in Central and South Americas and it has been translocated with the cane toad into the Australia but has not infected Australian endemic frog species [17]. Translocation of a multi-host parasite with the cane toad has yet to be unambiguously demonstrated. Host specificity is a fundamental property of all parasites, and parasite faunas of invasive species consist of a mixture of authentic parasites translocated from its original range and those newly encountered parasites that had broad enough specificity to take the advantage of the invasive host [18].

Almost all we know about myxosporean parasites comes from their investigation in fish. Out of more than 2200 species, about 2000 are fish Myxosporea, of which we know the life-cycle of only 33. The parasite alternates between vertebrate and invertebrate hosts; asexual development occurs in vertebrate hosts leading to the production of resistant myxospores, and sexually in the invertebrate host leading to the production of fragile aquatic actinospores [6,13]. The introduction of a myxosporean parasite into Australian frogs from the cane toad has been assumed based on morphological evidence from extant frogs and the absence of written records of such gallbladder parasite in scientific records [7]. However, the two-host life cycle of Myxosporea and the unknown specificity for either vertebrate or invertebrate hosts has prompted us to re-examine this hypothesis using museum material. Indeed, the absence of Myxosporea in the gallbladders in frogs collected and catalogued prior to the introduction of the cane toad supports a recent emergence of the parasite. It is further supported by the absence of Myxosporea in the green and golden bell frog gallbladders from New Caledonia, where it is the only frog introduced in late 19^th ^century. However, does this ultimately victimise the cane toad? First, we do not know the life cycle of the parasite, or if it requires an invertebrate host. The majority (possibly all) of myxosporean species, including the pathogenic *Myxobolus cerebralis *requires its aquatic oligochaete host being infected by myxospores produced in salmonid fish. The success behind the global distribution of *Myxobolus cerebralis *is in the cosmopolitan distribution of its dominant invertebrate host *Tubifex tubifex *and its transcontinental trade [6]. However, direct transmission, fish-to-fish, was documented for several *Enteromyxum *species [19]. We know nothing about the life cycle in frogs, but it is likely that *Myxidium immersum *uses an invertebrate host in its lifecycle, and therefore international trade may also be implicated in *Myxidium immersum *distribution across continents similar to the spread of the chytrid fungus [4]. *Myxidium immersum *is described as a species with an extremely broad host specificity [7,15]; however, what might seem like a single morphospecies may mask a cryptic species complex for which molecular methods may resolve this difficulty [20]. We are currently collecting material to genetically characterise extant isolates from Australian frogs (AH, DNP, JŠ unpublished data).

## Conclusion

While the cane toad may appear as a prime suspect, the deleterious effects of successive cane toad translocations seems unfavourable to a parasite with a two host life cycle such as *Myxidium immersum *due to its complexity. The low prevalence (7%) in the museum material suggests that only 7 animals would be *Myxidium-*positive when first translocated into Australia, because the founding cane toad population was only 101 individuals [8]. If, indeed these were the founding animals for the dispersal of the parasite, than *Myxidium immersum *is one of the luckiest parasites in the world because it is found flourishing in > 20 endemic and exotic frog species and had even reached a distribution beyond the current cane toad range. The origin of the parasite needs to be considered further with regards to its status in the source population, an introduction with other frogs brought to Australia for laboratory purposes and accidental introduction via infected invertebrate hosts.

## Methods

### Voucher museum material

A catalogue of the Australian Museum, Sydney was queried for the green and golden bell frog (*Litoria aurea*), the green tree frog (*Litoria caerulea*), the Peron's tree frog (*Litoria peronii*), the striped marsh frog (*Limnodynastes peronii*), and the introduced cane toad (*Bufo marinus*, syn. *Rhinella marina*). Vouchers were originally fixed in ethanol or formalin for an unknown period of time prior to transfer into 70% ethanol. In total, 256 voucher specimens and their catalogue records (denoted by AM#) were recovered, including all specimens prior to 1935 (Additional file 1). A selection of museum specimens of the green and golden bell frog from New Caledonia was included (*n *= 29).

### Extraction of frog gallbladders

A small incision (0.5-1 cm) on the belly of the frog voucher was used to reach the gallbladder and preserve the integrity of the specimen. For sufficiently large specimens the content of the gallbladder was aspirated with a disposable G22-needle. For smaller specimens the entire gallbladder was removed. The individual gallbladder contents and the gallbladder were stored in 70% ethanol until examination or processing.

### Examination of gallbladders for myxospores

For a wet mount examination the entire gallbladder contents were examined using 20 × and 40 × objectives. Myxospores were measured/photographed using a 100 × objective BX51 microscope equipped with a DP70 camera (Olympus Australia); a minimum of 10 myxospores from each individual was measured and morphological details evaluated. Myxospores were prepared for scanning electron microscopy; fixed in 2% OsO_4 _in 0.2 M sodium cacodylate buffer (pH 7.0), rinsed in distilled water, dehydrated with a graded acetone series, critical point dried, coated with gold and examined using a Zeiss ULTRA *plus *FE. Individual gallbladders were selected for paraffin embedding, sectioning, Giemsa staining and examined for the presence of myxospores.

### Statistical analysis

The probability of observing no positive samples in the total number of samples collected was calculated using FreeCalc v2, an epidemiological probability calculator assisting with the planning and analysis of surveys to demonstrate freedom from disease [21]; http://www.ausvet.com.au/content.php?page=software - distributed by AusVet Animal Health Services.

We assumed 100% specificity of our detection of myxospores, because it relied on microscopic of the whole gallbladder content coupled with histology and Giemsa staining. We calculated that 57, 29 and 14 animals (p = 0.05) is the minimum number of animals to be taken from a population with negative result to consider the population is free from disease using 5%, 10% and 20% minimum expected prevalence, respectively. We also evaluated a conservative 90% test sensitivity yielding 66, 32 and 15 animals (p = 0.05) as the minimum number be taken from a population with negative result to consider the population is free of the disease expecting minimum prevalence of 5%, 10% and 20%, respectively.

The minimum expected prevalence is the lowest level of prevalence that we aimed to detect. Delvinquier (1986) detected the parasite in 50% (6/12) specimens of the striped marsh frog (*Limnodynastes peronii*), 46% (5/11) specimens of the green tree frog (*Litoria caerulea*), 42% (8/19) specimens of the Peron's tree frog (*Litoria peronii*), and 13/34 (38%) specimens of the cane toad (*Bufo marinus*); the green an golden bell frog (*Litoria aurea*) was not surveyed [7]. Despite these numbers we restricted ourselves to a conservative 5% minimum prevalence. In other words, the minimum expected prevalence means that if parasite were to be present below 5% level, our survey would not be able to detect it. This minimum expected prevalence was considered sufficient, because (i) Delvinquier (1986) [7] has detected much higher prevalence in Australian frogs and (ii), we calculated 13% (7/60) prevalence using our survey of museum frogs from 1975 onwards.

## Competing interests

The authors declare that they have no competing interests.

## Authors' contributions

All authors contributed to this study. JŠ and AH designed the study. AH performed the experiments and collected data. AH and JŠ analysed the data. JŠ, AH and DNP drafted the manuscript. All authors read and approved the final manuscript.

## Supplementary Material

Additional file 1 **Summary of the Australian Museum frog specimens and presence of *Myxidium *cf. *immersum***. Click here for file
