# Supplementary material for: Museum material reveals a frog parasite emergence after the invasion of the cane toad in Australia
Source: Parasit Vectors. 2010 Jun 10;3:50. doi: 10.1186/1756-3305-3-50 (PMC2901343; doi:10.1186/1756-3305-3-50)
Supplement: Additional file 1 — Summary of the Australian Museum frog specimens and presence of Myxidium cf. immersum. [file 1756-3305-3-50-S1.PDF]

Additional file 1. Summary of the Australian Museum (AM) frog specimens and presence of *Myxidium* cf. *immersum* .

Green and golden bell frog (*Litoria aurea*) (n=61)			
**AM#**	**Date**	**Location**	**Result**
19424	1890	Richmond, NSW	Negative
19425	1890	Richmond, NSW	Negative
4194	1908	Maroubra, NSW	Negative
4195	1908	Maroubra, NSW	Negative
4196	1908	Maroubra, NSW	Negative
4198	1908	Maroubra, NSW	Negative
4199	1908	Maroubra, NSW	Negative
4200	1908	Maroubra, NSW	Negative
4201	1908	Maroubra, NSW	Negative
4202	1908	Maroubra, NSW	Negative
4203	1908	Maroubra, NSW	Negative
4205	1908	Maroubra, NSW	Negative
4208	1908	Maroubra, NSW	Negative
4251	1908	Nambucca, NSW	Negative
4665	1910	Woonona, NSW	Negative
4666	1910	Woonona, NSW	Negative
4667	1910	Woonona, NSW	Negative
4668	1910	Woonona, NSW	Negative
5389	1^	North Sydney, NSW	Negative
7446	1^	Pambula, NSW	Negative
7974	1^	Upper Colo, NSW	Negative
8454	1^	Unknown	Negative
8456	1^	Sydney, NSW	Negative
8483	1^	Sydney, NSW	Negative
18464	1^	Wollongong, NSW	Negative
18768	1^	Shoalhaven Heads, NSW	Negative
19428	1^	Hillgrove, NSW	Negative
19669	1958	Singleton, NSW	Negative
19670	1958	Singleton, NSW	Negative
19671	1958	Singleton, NSW	Negative
19672	1958	Singleton, NSW	Negative
19674	1958	Singleton, NSW	Negative
19675	1958	Singleton, NSW	Negative
19676	1958	Singleton, NSW	Negative
19677	1958	Singleton, NSW	Negative
19678	1958	Singleton, NSW	Negative
19679	1958	Singleton, NSW	Negative
19680	1958	Singleton, NSW	Negative
19681	1958	Singleton, NSW	Negative
144869	1980	Royal National Park, NSW	Negative
146888	1994	Homebush, NSW	Negative
148744	1996	Homebush, NSW	Positive
148745	1996	Homebush, NSW	Negative
148746	1996	Homebush, NSW	Negative
149255	3^	Unknown	Negative
150422	3^	Breadalbane, NSW	Negative
150424	3^	Breadalbane, NSW	Negative
150425	3^	Port Stephens, NSW	Negative
150429	3^	Somersby, NSW	Negative
150994	1997	Rosebery,NSW	Negative
151330	1997	Rosebery,NSW	Negative
151994	1997	Rosebery,NSW	Negative
153967	1997	Homebush, NSW	Positive
155970	1997	Homebush, NSW	Negative
159000	1993	Rosebery,NSW	Negative
166405	1974	Smiths Lake, NSW	Negative
166407	1974	Smiths Lake, NSW	Negative
166408	1974	Smiths Lake, NSW	Negative
170186	3^	Crescent Head, NSW	Negative
171115	3^	captive reared	Negative
175438	3^	Unknown	Negative

Green and golden bell frog (*Litoria aurea*) NEW CALEDONIA (n=29)			
**AM#**	**Date**	**Location**	**Result**
165857	2003	Mt. Aoupinie, New Caledonia	Negative
165858	2003	Mt. Aoupinie, New Caledonia	Negative
165859	2003	Mt. Aoupinie, New Caledonia	Negative
165860	2003	Mt. Aoupinie, New Caledonia	Negative
165861	2003	Mt. Aoupinie, New Caledonia	Negative
165862	2003	Mt. Aoupinie, New Caledonia	Negative
165863	2003	Mt. Aoupinie, New Caledonia	Negative
166009	2003	Plaine Des Lacs, New Caledonia	Negative
166026	2003	Plaine Des Lacs, New Caledonia	Negative
166109	2003	Plaine Des Lacs, New Caledonia	Negative
166110	2003	Plaine Des Lacs, New Caledonia	Negative
166115	2003	Plaine Des Lacs, New Caledonia	Negative
166139	2003	Plaine Des Lacs, New Caledonia	Negative
166141	2003	Plaine Des Lacs, New Caledonia	Negative
166174	2003	Plaine Des Lacs, New Caledonia	Negative
166175	2003	Plaine Des Lacs, New Caledonia	Negative
166177	2003	Plaine Des Lacs, New Caledonia	Negative
166178	2003	Plaine Des Lacs, New Caledonia	Negative
166179	2003	Plaine Des Lacs, New Caledonia	Negative
166180	2003	Plaine Des Lacs, New Caledonia	Negative
167109	2005	Bopope, New Caledonia	Negative
167110	2005	Bopope, New Caledonia	Negative
167111	2005	Bopope, New Caledonia	Negative
167112	2005	Bopope, New Caledonia	Negative
167113	2005	Bopope, New Caledonia	Negative
167114	2005	Bopope, New Caledonia	Negative
167115	2005	Bopope, New Caledonia	Negative
167117	2005	Bopope, New Caledonia	Negative
167118	2005	Bopope, New Caledonia	Negative

Green tree frog (*Litoria caerulea*) (n=60)			
**AM#**	**Date**	**Location**	**Result**
1899	1895	Sydney,NSW	Negative
3538	1903	Mapoon, QLD	Negative
3833	1905	Sydney,NSW	Negative
4289	1908	Sydney,NSW	Negative
4290	1908	Sydney,NSW	Negative
4292	1908	Darling Floods, NSW	Negative
4293	1908	Darling Floods, NSW	Negative
4294	1908	Darling Floods, NSW	Negative
4520	1909	Murray Island, Torres Strait	Negative
4522	1909	Murray Island, Torres Strait	Negative
4524	1909	Murray Island, Torres Strait	Negative
4614	1909	New Guinea	Negative
5088	1909	Sydney,NSW	Negative
5092	1911	Sydney,NSW	Negative
5177	1911	Sydney,NSW	Negative
5243	1911	Darling Floods, NSW	Negative
5245	1911	Darling Floods, NSW	Negative
5355	1911	Murray Island, Torres Strait	Negative
5437	1911	Lismore, NSW	Negative
6115	1913	Unknown	Negative
6328	1913	Eidsvold, QLD	Negative
6329	1913	Eidsvold, QLD	Negative
6330	1913	Eidsvold, QLD	Negative
6522	1913	Central Northern Queensland	Negative
6523	1913	Central Northern Queensland	Negative
6524	1913	Central Northern Queensland	Negative
6648	1914	Herbert River, QLD	Negative
6649	1914	Herbert River, QLD	Negative
6984	1917	Kimberleys, W.A	Negative
7336	1921	Goangra, NSW	Negative
7337	1921	Goangra, NSW	Negative
7338	1921	Goangra, NSW	Negative
7339	1921	Goangra, NSW	Negative
7464	1922	Dunoon, NSW	Negative
7594	1^	Unknown	Negative
9680	1928	Murray Island, Torres Strait	Negative
9721	1929	Groote Eylandt, NT	Negative
9722	1929	Groote Eylandt, NT	Negative
11021	1933	Groote Eylandt, NT	Negative
11053	1933	Moree, NSW	Negative
11070	1933	Wyong, NSW	Negative
11154	1934	Tyalgum, NSW	Negative
11161	1934	Lindeman Island, Whitsundays	Negative
11593	1935	Pillaga, NSW	Negative
11594	1935	Pillaga, NSW	Negative
11595	1935	Pillaga, NSW	Negative
11706	1935	Lindeman Island, Whitsundays	Negative
11781	1^	Nyngan, NSW	Negative
12400	2^	Yirrkala, NT	Negative
12401	2^	Yirrkala, NT	Negative
12402	2^	Yirrkala, NT	Negative
12403	2^	Yirrkala, NT	Negative
13125	2^	Tenterfield, NSW	Negative
13126	2^	Tenterfield, NSW	Negative
13645	1948	Cape Arnhem, NT	Negative
14802	2^	Bowen, QLD	Negative
15320	2^	Maclean, NSW	Negative
15708	1959	Goonoo State Forest, NSW	Negative
99574	1966	Wallacia, NSW	Positive
99575	1966	Wallacia, NSW	Positive

Peron's tree frog *(Litoria peronii)* (n=16)			
**AM#**	**Date**	**Location**	**Result**
3938	1^	Unknown	Negative
4295	1908	Darling River Floods, NSW	Negative
4663	1910	Woonona, NSW	Negative
5443	1^	Buddah Lake, NSW	Negative
7528	1^	ClarenceRiver, NSW	Negative
7529	1^	ClarenceRiver, NSW	Negative
7964	1^	NSW	Negative
8092	1^	Buladelah, NSW	Negative
8423	1^	Mount Horeb, NSW	Negative
9396	1^	Unknown	Negative
10690	1932	Yanco, NSW	Negative
10691	1932	Yanco, NSW	Negative
10693	1932	Yanco, NSW	Negative
10695	1932	NSW	Negative
10696	1932	Yanco, NSW	Negative
10697	1932	Yanco, NSW	Negative

Striped marsh frog (*Limnodynastes peronii*) (n=42)			
**AM#**	**Date**	**Location**	**Result**
1879	1^	Waverley, NSW	Negative
1880	1^	Waverley, NSW	Negative
1881	1^	Waverley, NSW	Negative
1882	1^	Waverley, NSW	Negative
4333	1909	Killara, NSW	Negative
4336	1909	Killara, NSW	Negative
4338	1909	Killara, NSW	Negative
4360	1909	Killara, NSW	Negative
4382	1909	Killara, NSW	Negative
4383	1909	Killara, NSW	Negative
4384	1909	Killara, NSW	Negative
4387	1909	Killara, NSW	Negative
4389	1909	Killara, NSW	Negative
4481	1909	Lindfield, NSW	Negative
4482	1909	Lindfield, NSW	Negative
4483	1909	Lindfield, NSW	Negative
4484	1909	Lindfield, NSW	Negative
4485	1909	Lindfield, NSW	Negative
4736	1910	Darling Flood Plains, NSW	Negative
4739	1^	NSW	Negative
5201	1^	Katoomba, NSW	Negative
7496	1^	Richmond River, NSW	Negative
9433	1^	Wentworthville, NSW	Negative
9946	1929	East Dorrigo, NSW	Negative
12035	1937	Lindfield, NSW	Negative
15170	1954	Burrawang, NSW	Negative
15452	1959	Mt.Wilson, NSW	Negative
166523	1965	Rules Point, ACT	Negative
156242	1999	Narooma, NSW	Negative
158418	3^	Brennan's Creek, NSW	Positive
158460	3^	Unknown	Negative
158462	3^	Unknown	Negative
161800	1995	Olney State Forest, NSW	Negative
161801	1995	Sweetmans Creek, NSW	Negative
162436	2002	Taree, NSW	Negative
162439	2002	Taree, NSW	Negative
162439	2002	Taree, NSW	Positive
162440	2002	Taree, NSW	Negative
162444	2002	Taree, NSW	Negative
162447	2002	Taree, NSW	Negative
170167	3^	Unknown	Positive
170178	3^	Unknown	Negative

Cane toad (Bufo marinus, syn. *Rhinella marina*) (n=48)			
**AM#**	**Date**	**Location**	**Result**
14306	2^	Queensland	Negative
16373	1960	South Johnstone, QLD	Negative
16374	1960	South Johnstone, QLD	Negative
16376	1960	South Johnstone, QLD	Negative
16377	1960	South Johnstone, QLD	Negative
16378	1960	South Johnstone, QLD	Negative
16384	1960	Sandy Creek, QLD	Negative
17110	2^	Lappa Junction, QLD	Negative
17111	2^	Lappa Junction, QLD	Negative
27614	1964	Longford Creek, QLD	Negative
27615	1964	Longford Creek, QLD	Negative
27616	1964	Longford Creek, QLD	Negative
27617	1964	Longford Creek, QLD	Negative
27618	1964	Gladstone, QLD	Negative
27619	1964	Gladstone, QLD	Negative
27622	1964	Gladstone, QLD	Negative
27623	1964	Gladstone, QLD	Negative
27624	1964	Gladstone, QLD	Negative
27625	1964	Gladstone, QLD	Negative
27628	1964	Gladstone, QLD	Negative
56441	2^	Hilda Creek, QLD	Negative
59918	1953	Mundubera, QLD	Negative
59919	1963	Gladstone, QLD	Negative
59920	1963	Gladstone, QLD	Negative
59921	1963	Gladstone, QLD	Negative
59922	1967	Mary River, QLD	Positive
62794	2^	Palmwoods,QLD	Negative
63103	1977	Charters Tower, QLD	Negative
63104	1977	Charters Tower, QLD	Negative
107585	1982/3	Weipa, QLD	Negative
107830	1983	Weipa, QLD	Negative
107831	1983	Weipa, QLD	Negative
107832	1983	Weipa, QLD	Negative
107843	1983	Weipa, QLD	Negative
107844	1983	Weipa, QLD	Negative
107845	1983	Weipa, QLD	Negative
107853	1983	Weipa, QLD	Negative
107854	1983	Weipa, QLD	Negative
107855	1983	Weipa, QLD	Negative
107857	1983	Weipa, QLD	Negative
107866	1983	Weipa, QLD	Negative
107874	1971	Marlborough, QLD	Negative
107875	1971	Marlborough, QLD	Negative
107876	1971	Marlborough, QLD	Negative
107877	1971	Marlborough, QLD	Negative
140594	1992	Alstonville, NSW	Negative
147183	1995	Terry Hills, NSW	Negative
158500	1998	Burringar, NSW	Positive

Notes:

Note that some vouchers are only dated based on a submission date, due to absence of collection date in the Australian Museum records: 1^ collected from before 1935, 2^ collected from 1936 to 1975, and 3^ collected from 1975 to present.

AM# - catalogue number in the Australian Museum (Sydney, NSW, Australia)

NSW - New South Wales, ACT - Australian Capital Territory, QLD - Queensland
